# Disentangling motivation and engagement: Exploring the role of effort in promoting greater conceptual and methodological clarity

**DOI:** 10.3389/fpsyg.2022.1045717

**Published:** 2022-12-13

**Authors:** Robin P. Nagy, Andrew J. Martin, Rebecca J. Collie

**Affiliations:** School of Education, University of New South Wales, Sydney, NSW, Australia

**Keywords:** motivation, effort, engagement, academic development, validity, multilevel

## Abstract

Conflation over motivation and engagement has historically impeded research and practice. One reason for this is because definition and measurement have often been too general or diffuse—especially in the case of engagement. Recently conceptual advances aimed at disentangling facets of engagement and motivation have highlighted a need for better psychometric precision—particularly in the case of engagement. To the extent that engagement is inadequately assessed, motivation research involving engagement continues to be hampered. The present study investigates multidimensional effort (a specific facet of engagement) and how it relates to motivation. In particular, we examine the associations between specific positive and negative motivation factors and dimensions of effort, thereby shedding further insight into how different types of motivation interplay with different types of engagement. Drawing on data from a sample of 946 Australian high school students in 59 mathematics classrooms at five schools, this study hypothesized a tripartite model of academic effort in terms of operative, cognitive, and social–emotional dimensions. A novel nine-item self-report Effort Scale measuring each of the three factors was developed and tested for internal and external validity—including its relationship with multidimensional motivation. Multilevel confirmatory factor analyses were conducted to test the factor structure and validity of multidimensional effort. Additionally, doubly-latent multilevel structural equation models were conducted to explore the hypothesized motivation → engagement (effort) process, and the role of student- and classroom-level background attributes as predictors of both motivation and effort. Results supported the hypothesized model of tripartite effort and its distinctiveness from motivation, and showed that key dimensions of motivation predicted effort at student- and classroom-levels. This study provides implications and suggestions for future motivation research and theorizing by (1) establishing evidence for the validity of a novel engagement framework (multidimensional effort), and (2) supporting future measurement and practice in academic engagement juxtaposed with multidimensional motivation—critical for better understanding engagement, and motivation itself.

## Introduction

Motivation and engagement are two intertwined constructs that have a history of conflation by researchers and practitioners. This has at times impeded advances in theoretical clarity, research, and practice relevant to both constructs (Reschly and Christenson, 2012). For example, it has been highlighted that inappropriately conflating motivation and engagement can create theoretical ambiguity, introduce validity challenges for measurement and research, and lay a shaky foundation for educational intervention (Martin, 2012; Reschly and Christenson, 2012; Martin et al., 2017). In recent years, much theorizing and research has been conducted into the multidimensionality of motivation (e.g., Martin, 1999–2022) and engagement (e.g., Fredricks et al., 2004). However, the basic demarcation of motivation as intent, and engagement as action, has thus far limited a more nuanced understanding of unique associations between their various dimensions, particularly with respect to non-observable dimensions of engagement. Whereas reliable scales have been developed and extensively tested for the measurement of motivation’s key dimensions, there has been much less focus on theoretically-informed measurement of multidimensional engagement, especially its internal aspects. By harnessing such a measurement scale, motivation and engagement can be further disentangled by examining the relation between adaptive and maladaptive motivation factors and specific dimensions of effort, thereby shedding further insight into the interplay of different motivation and engagement types.

Effort (as a specific form of engagement) is an illustrative case in point of the blurred conceptual and empirical terrain relevant to engagement. Despite appearing ubiquitously throughout the engagement literature, it is as yet a largely untapped and undefined construct that warrants further attention and definition. In this study, we therefore closely considered effort from a conceptual perspective and harnessed this conceptual foundation to develop a multidimensional measure of it—the Effort Scale. In particular, it was anticipated that this novel tool would enable demarcation between individual motivation dimensions and their unique associations with different types of effort. Mathematics was chosen as a specific subject area of focus, due to well-documented declines in motivation and engagement highlighted by recent research (see Collie et al., 2019), together with continued declines in students’ mathematics achievement, especially in Australia (e.g., Thomson et al., 2016, 2019).

**Figure 1:**
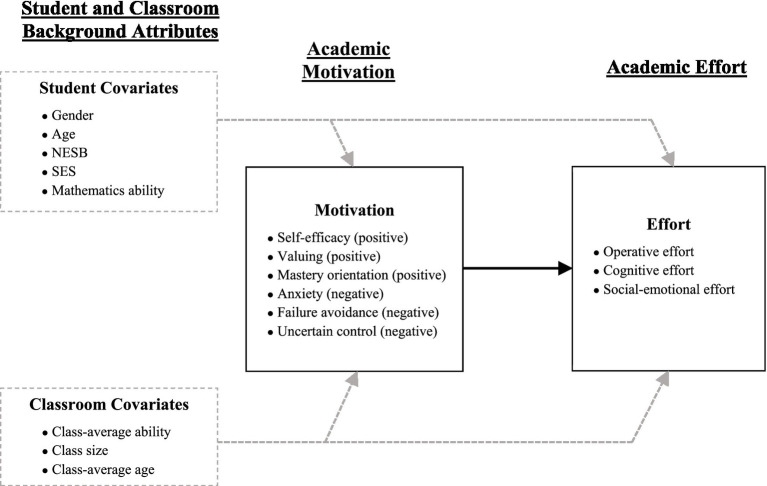
Hypothesized full process model (processes are estimated at student- and classroom-levels). SES, Social-economic status indicator; NESB, Non-English speaking background indicator.

Utilizing a multilevel approach, we tested the measurement properties of the Effort Scale at student- and classroom-levels in mathematics to determine its psychometric properties and its associations with multidimensional motivation *via* bivariate correlations at both levels. We then employed structural equation modelling to examine the role of multidimensional motivation in predicting multidimensional effort at student- and classroom-levels (as shown in Figure 1). Through these conceptual and empirical processes, we shed further light on the unique and shared variance between motivation and engagement (by way of effort) and provide a foundation for greater clarity and coherence for educational researchers and practitioners in their future work aimed at optimizing students’ academic outcomes.

## Motivation and engagement

To foreground our study of motivation and engagement, we first briefly summarize some key features of motivation and engagement, some broad dimensions that distinguish them, and the multidimensional motivation framework we harness as a means to better understand how motivation and engagement interrelate.

### Where have we been? Where are we now? Where are we going?

Motivation and engagement are significant areas of interest in educational psychology, seen as drivers of proximal and long-term academic (and other) success and accomplishment (Reschly and Christenson, 2012). Out of the two, motivation has received far more focused theorizing and research, as indicated by the numerous major theories that have been developed in the past five decades (e.g., social cognitive theory, Bandura, 2012; goal theory, Elliot, 2005; need achievement theory, McClelland, 1961; self-worth theory, Covington, 2000; self-determination theory, Ryan and Deci, 2017; [situated] expectancy-value theory, Wigfield and Eccles, 2000; Eccles and Wigfield, 2020; etc.). By contrast, there are few theories about engagement, and relatively little work on clarifying its measurement and theoretical grounding. However, in the past 2 decades, there has been an uptick in attention being given to engagement.

There is now broad consensus that engagement is multidimensional, comprising components of behavior, cognition, and emotion/affect (Fredricks et al., 2004), but there remain differing ideas about how these dimensions are defined and where they reside within an overarching “engagement” construct (Christenson et al., 2012). In their review of student engagement, Reschly and Christenson (2012) identified three main channels of engagement literature: one driven by reducing school dropout, one emanating from a school reform perspective, and one emerging from motivation theory and research. Especially in relation to the latter, there has been conflation with motivation theory, definitions, and measurement. Given the lack of consensus on definitions of engagement and its association with/distinction from motivation, Reschly and Christenson (2012) argued there is a need for theoretical and psychometric advancement of engagement that can then be implemented in motivation research in order to better understand the two. They encouraged new expositions of engagement to advance the field, and to test the convergent and divergent validity of these expositions in relation to motivation. With a focus on multidimensional effort, the present study offers one approach toward a new exposition of multidimensional engagement, and its alignments and differences from motivation. The envisaged yields are 2-fold: better understanding and measurement of engagement (*via* a novel multidimensional effort framework and measurement tool) that affords a better understanding of the unique associations between motivation’s and engagement’s individual dimensions.

### Differentiating motivation and engagement

As noted, in the past 2 decades, researchers have attended more closely to the distinctions and alignments between motivation and engagement. In his commentary on major researchers’ perspectives on motivation and engagement, Martin (2012) (see also Martin, 2022) observed that at a fundamental level, motivation and engagement may be demarcated into internal and external dimensions. For example: Reeve (2012) suggested motivation comprises “private, unobservable, psychological, neural, and biological” factors, while engagement constitutes “publicly observable behavior” (p. 151); Ainley (2012) identified motivation in terms of inner psychological factors, whereas engagement reflected more outward involvement; and, Voelkl (2012) suggested that motivation aligns with internal affective states and engagement with behavioral factors. All this being the case, motivation has been defined as the inclination, energy, emotion, and drive to learn, work effectively, and achieve—and engagement as the more externally-evident factors reflecting the internal motivational phenomena (e.g., Martin et al., 2017). However, although helpful in clearly differentiating between these two constructs, this basic demarcation of motivation as internal, versus engagement as external, is not intended as a prescriptive or definitive distinction.

Many researchers illustrate the blurred edges to this internal/external classification, referring to internal facets of engagement, typically characterized by cognitive and affective/emotional dimensions (e.g., Fredricks et al., 2004; Appleton et al., 2006; Cleary and Zimmerman, 2012; Wang and Eccles, 2012; Morgan et al., 2022). Indeed, Reschly and Christenson (2012) specifically highlight conflation over these internal facets of engagement and aspects of motivation, such as self-regulation. They point out that defining motivation as intent (internal), and engagement as action (external), implies that engagement is always behavioral and so observable, whereas it is clear that cognitive and affective engagement are largely internal processes, and so apparently indistinguishable from motivation using this distinction.

As such, we draw on the definitions of motivation and engagement in Martin et al. (2017) and extend them for this study, with motivation being the inclination and drive to learn, work effectively, and achieve—and engagement as the expression of this inclination and drive to learn *via* either external (e.g., behaviors) or internal (e.g., cognitive and affective) processes. In this study, we aim to expand this distinction between the two constructs by capturing a more comprehensive engagement characterization (targeting effort as a specific active form of engagement). Our study encompasses both internal and external dimensions of engagement, and specifically distinguishes between its internal aspects (e.g., cognitive and social–emotional) and motivation.

In addition to motivation being considered as an internal process, and engagement as both internal and external, there is tentative agreement about the ordering of the process in which they manifest, with motivation generally considered to lead to engagement. For example, Schunk and Mullen (2012) used social-cognitive theory as the basis for explaining how motivation and engagement inter-relate, with self-efficacy (a motivation factor) influencing behavioral engagement. In another conceptualization, Cleary and Zimmerman (2012) employed self-regulation theory to describe how self-efficacy (motivation) leads to changes in engagement (encompassing strategizing and self-regulatory processes). There is thus some agreement that “motivation is a basis for subsequent engagement” (Martin, 2012, p. 305). This hypothesized ordering of motivation and engagement is important in the present study as it is a means to examine how motivation and engagement inter-relate and is thus a way to better understand both constructs. Specifically, we investigated the extent to which multidimensional motivation predicted a novel engagement (effort) construct (see Figure 1).

### Multidimensional motivation

The Motivation and Engagement Wheel (Martin, 1999–2022) has been developed to capture multidimensional motivation as proposed by seminal motivation theorizing. It is the framework harnessed in the present study as the means to better understand how motivation and engagement (by way of multidimensional effort) interrelate. The Wheel comprises six (first order) motivation factors that can also be integrated to form two higher-order factors (positive/adaptive and negative/maladaptive motivation). Positive motivation consists of: self-efficacy (the belief and confidence in one’s ability to learn), valuing (the belief in the importance, usefulness, and relevance of one’s academic work), and mastery orientation (the orientation to develop one’s learning and task mastery). Negative motivation comprises: anxiety (the tendency to feel anxious about one’s academic work), failure avoidance (the inclination to work in order to avoid doing poorly), and uncertain control (the lack of agency in effecting positive academic outcomes). Positive motivation factors reflect students’ positive attitudes and orientations to academic learning, whereas negative motivation factors represent students’ attitudes and orientations that inhibit learning. As noted, these six factors emanate from foundational motivation theories. Self-efficacy is very much based on the work of Bandura (2001) and reflects students’ task-specific competence beliefs. Valuing draws on (situated) expectancy-value theory (Wigfield and Eccles, 2000; Eccles and Wigfield, 2020) that underscores the motivational boost students experience if they value a task in one or more ways (e.g., in terms of utility and importance). Mastery orientation is underpinned by goal theory (Elliot, 2005), which reflects students’ goal orientation towards achieving academic success *via* effort, skill development, and learning. Anxiety and failure avoidance draw from need achievement and self-worth theories (McClelland, 1961; Covington, 2000) that offer perspectives on students’ fear of failure (failure avoidance is also implicated in goal theory by way of performance avoidance goals; Elliot, 2005). Finally, uncertain control is informed by attribution theory (Weiner, 2010) which describes how the dimensions of stability, locus, and control influence students’ motivation to learn.

The factors in the Wheel are assessed *via* an accompanying assessment tool, the Motivation and Engagement Scale (MES; Martin, 1999–2022). The MES has been extensively employed and tested in a variety of research studies (see Liem and Martin, 2012 for a review). The MES assesses not only the six positive and negative motivation factors described above, but also three positive engagement factors (planning and monitoring, task management, and persistence) and two negative engagement factors (self-handicapping and disengagement). With an expanded definition of engagement (by way of effort) that includes internal as well as external factors, the present study extends the operationalization of engagement in the MES to engagement factors outside it. To our knowledge, only one study has investigated the predictive links between the MES motivation and engagement factors, tentatively suggesting that motivation predicts engagement (Martin et al., 2017). The present study’s focus on multidimensional effort (as an active, energetic form of positive engagement), and how the six motivation factors predict it (Figure 1), is an opportunity to incorporate a new measure of engagement into the evidence base.

### Multidimensional effort: A means to better understand engagement and motivation

Researchers are increasingly focusing on students’ engagement at school as a predictor of academic success (Lei et al., 2018). Fredricks et al. (2004) provided a seminal review of research and theorizing on engagement, describing it in terms of a (multidimensional) tripartite model, with behavioral, cognitive, and emotional engagement as constituent factors of an overarching engagement construct. Fredricks et al. (2004) described behavioral engagement in terms of student involvement and participation in school activities (in both academic and non-academic arenas). They described cognitive engagement in terms of a willingness and thoughtfulness to invest effort to comprehend academic concepts. Emotional engagement was described as encompassing (both positive and negative) reactions to teachers, peers, and the school environment (thus, also reflecting a social–emotional element), that in turn influences students’ willingness to invest effort. Engagement is thus now generally acknowledged to be a multidimensional construct, typically considered as tripartite with behavioral, cognitive, and affective (or emotional) components (e.g., Christenson et al., 2012; Lei et al., 2018).

### Tripartite effort

Of particular relevance to the present investigation, the meta-analysis of engagement and achievement conducted by Lei et al. (2018), framed students’ tripartite engagement in terms of being actively involved in learning tasks and learning processes. Active engagement implies the investment of energy and effort in learning tasks, as opposed to a more passive involvement in class (such as passively watching a video, or listening/paying attention in class but not making any effort to participate or play an active role in discussions). This emphasis on active (as opposed to passive) engagement implies effortful engagement in each of the constituent tripartite engagement dimensions. This being the case, we propose that academic effort sits under the umbrella construct of (positive) engagement and comprises similar components, namely: behavioral, cognitive, and affective/emotional dimensions—with a higher-order effort factor that represents the theoretical and empirical confluence of these first-order dimensions.

The few researchers who have sought to more explicitly account for both engagement and effort have emphasized the importance of distinguishing between them. For example, it is clear from Fredricks et al. (2004) that whereas school engagement encompasses positive and negative (e.g., disengagement) academic and non-academic dimensions, academic effort is a sub-component of school engagement that specifically relates to the academic arena and involves positive engagement (not disengagement) that is an active, volitional expenditure of energy in the domains of behavior, cognition, and social–emotional interactions. Fredricks et al. (2004) acknowledged that although engagement has received substantial empirical attention, it is theoretically messy and overlaps considerably with other constructs. According to them, the broad umbrella term of “engagement” is problematic as “it can result in a proliferation of constructs, definitions, and measures of constructs that differ slightly, thereby doing little to improve conceptual clarity” (p. 60). Of relevance to the present study, Fredricks et al. identified effort (a construct incorporated under engagement) as a particular example of this, and an avenue requiring further clarification and then investigation in this space. Indeed, Fredricks et al. (2004), Nagy (2016, 2017), and Carbonaro (2005) have all underscored the importance of effort and its multidimensional nature, comprising behavioral (or operative), cognitive, and social–emotional factors.

Following Fredricks et al. (2004), Nagy (2016, 2017), and Carbonaro (2005), the behavioral dimension of effort in the present study is focused on the notion of “doing” and “outcomes-completion”—referred to herein as *operative effort* and defined as active, purposeful, and energetic action-based application to learning. Operative effort is typified by the application of behavioral energy in the production and completion of schoolwork. *Cognitive effort* is defined as active, purposeful, and energetic mental/psychological application to learning. It is typified by concentration, attention, and focus directed toward understanding, comprehension, and mastery of schoolwork. *Social–emotional effort* is defined as active, purposeful, and energetic interpersonal/affective application to learning. It is typified by appropriate and respectful classroom social–emotional interactions that involve self-control and sensitivity to the social context of learning, conducive to completing schoolwork.

### Measurement of tripartite effort

Building on this tripartite framing of effort in terms of its operative, cognitive, and social–emotional dimensions, a multidimensional effort scale (hereafter, the Effort Scale) was developed for implementation in the present study. This Effort Scale is designed to capture the three distinct aspects of effort, and also to represent a hypothesized overarching effort factor reflecting appropriate weighting (or loading) of each of the three constituent factors onto the whole—enabling both specificity (in the case of a first-order structure) and broader application (in the case of a higher-order structure) as appropriate to the research purpose. It is this tool that will represent an approach to multidimensional engagement (i.e., *via* effort) and be the basis of analyses with multidimensional motivation in the present study. It is described more fully in the section Materials and methods, below.

### Context and background attributes relevant to motivation and engagement

In line with major motivation theories (e.g., Bandura, 2001; Ryan and Deci, 2017; Eccles and Wigfield, 2020), we also accounted for contextual and background attributes known to be implicated in motivation and engagement. We did so in two ways: by employing multilevel modelling to extend the typical student-level analyses of motivation and engagement to analyses at the classroom-level, and by including numerous pertinent student- and classroom-level background attributes as predictors of motivation and engagement (see Figure 1). The former enabled us to disentangle student- and classroom-level motivation and engagement. The latter enabled us to determine the unique association of motivation predicting effort, by controlling for variance attributable to pertinent student- and classroom-level background factors.

## Aims of the present study

Historical conflation over the intertwined constructs of motivation and engagement has impeded advances in theory and practice relating to students’ academic development. As the field of engagement has progressed over the past decade, advances in its theoretical conceptualizing have led to new opportunities to better understand the interface between motivation and engagement. Questions can now be posed that further unpick the distinctiveness of these two intertwined constructs, such as how positive and negative motivation factors uniquely predict individual dimensions of engagement. This study seeks to bring clarity to this space through a purposeful investigation using the hitherto untapped and undefined construct of effort (representing a specific active form of multidimensional engagement) and to investigate the unique associations of its respective dimensions with multidimensional motivation. We aimed to closely consider effort from a conceptual perspective, hypothesizing a tripartite model of academic effort in terms of operative, cognitive, and social–emotional dimensions—and then developing a practical multidimensional effort measure—the Effort Scale, incorporating each of the three component factors.

We adopted a construct validation approach to explore motivation and this novel effort framework (e.g., Marsh, 1997, 2002; Martin and Marsh, 2008). Such an approach considers assessment of the validity of both within-network (“internal validity”) and between-network (“external validity”). We pursued this construct validation by first testing the measurement properties of the Effort Scale at student- and classroom-levels (internal validity), then testing the association between motivation and effort *via* bivariate correlations at both levels (external validity), and then examining the role of motivation predicting effort at student- and classroom-level (external validity), appropriately controlling for pertinent student- and classroom-level background attributes (see Figure 1). For the purposes of this study, we are particularly interested in convergent (the extent to which motivation is associated with effort in theoretically plausible ways) and discriminant (the extent to which there remains sufficient unshared variance to indicate their distinctiveness) aspects of the constructs’ external validity. We hypothesized that motivation and effort would be associated with each other (by way of correlations and predictive parameters) but left as an open empirical question the precise nature and strength of associations between their different dimensions.

## Materials and methods

### Participants and procedure

The sample for this study comprised 946 Australian high school students nested within 59 mathematics classrooms from five schools. The sample was chosen, within the given constraints and practicalities of data collection, to be as diverse as possible in terms of gender, academic ability, age, and school gender profile (*viz.* single-sex or coeducational) and therefore as representative as possible of potentially influential covariate attributes. All schools were non-academically selective in intake, in the independent school sector and located in and around a major capital city of New South Wales (NSW) on the east coast of Australia. Of the five schools, three were coeducational, one was a single-sex boys’ school, and one was a single-sex girls’ school. Just over half (53%) of students were boys. Students were in the first 4 years of high school in Australia and comprised: Year 7 (8%), Year 8 (41%), Year 9 (34%), and Year 10 (17%). The average age was 14.70 years (*SD* = 0.98 years). Non-English-speaking background (NESB) students accounted for 16% of the sample. Students typically came from higher socio-economic status (SES) postal districts (*M* = 1,084, range from 846 to 1,179, *SD* = 64) than the Australian average (*M* = 1,000, *SD* = 100) based on the Australian Bureau of Statistics (ABS) index of relative socio-economic advantage and disadvantage classification (SEIFA; Australian Bureau of Statistics, 2016). Of the 59 classrooms, class size varied from 7 to 29 (*M* = 21, *SD* = 5), with participation rates ranging from 31% to 100% (*M* = 74%, *SD* = 17%). Human ethics approval was received from the lead researcher’s university, and school principals then provided approval for their school’s participation in the study. Following this, parents/careers and students provided consent. An online survey was administered to students, in a regular mathematics lesson, in the final term of 2020.

### Materials

The measures included in the survey comprised the substantive factors of motivation and effort. We also assessed student and classroom background attributes as covariates.

#### Motivation

Motivation was measured using six self-rated items from the brief form of the Motivation and Engagement Scale—High School [MES-HS-Short; Martin, 1999–2022]. The items captured three positive motivation constructs (self-efficacy, valuing, and mastery orientation) and three negative motivation constructs (anxiety, failure avoidance, and uncertain control). The items (e.g., for self-efficacy, “I believe I can do well in this subject”) were rated using a seven-point Likert scale (1 = *strongly disagree* to 7 = *strongly agree*). As each motivation factor was represented by a single-item, we could not estimate them as latent variables, and so we modelled each factor as error-adjusted mean scores so that our analyses could correct for unreliability (as latent modeling would do). The following equation was used to calculate the error-adjusted mean score: σ^2 *^ (1−ω), where σ^2^ is the estimated variance of the substantive factor and ω is the reliability estimate of this factor (Hayduk, 1987; see also Cole and Preacher, 2014). The reliabilities (omega total; McNeish, 2018) and variances were taken from a prior research program using the full (multi-item) MES-HS in mathematics (Martin and Marsh, 2005; Marsh et al., 2008). Descriptive statistics for the present study are presented in Results.

#### Academic effort

Student-rated effort was measured using a three-factor, nine-item scale (the Effort Scale) emanating from work by Nagy (2016, 2017, 2022). Operative effort was measured *via* three items [e.g., “In mathematics, I try hard on schoolwork (e.g., in class or at home etc.) given to me”]; cognitive effort was measured *via* three items (e.g., “I am focused in mathematics class”), and social–emotional effort was measured *via* three items [e.g., “I show self-control in mathematics lessons (e.g., I wait my turn, do not interrupt, and do not talk over other students etc.)”]. As described in the Introduction, the hypothesized effort framework comprises three first-order factors and also an overarching higher-order effort factor. All effort items were rated using a seven-point Likert scale (1 = *strongly disagree* to 7 = *strongly agree*) and are detailed in Supplementary material (Supplementary Table S1). For completeness, also presented in Supplementary material is a brief form of the Effort Scale (the Effort Scale—Short [ES-S]; one item for each of the three dimensions, thus a three-item measure)—and its psychometric properties and correlations with motivation. Descriptive, reliability, and factor analytic findings for first- and higher-order Effort Scale factors are presented in the Results section below.

#### Student and classroom background attributes

Our hypothesized process model (Figure 1) was designed to assess the unique associations between motivation and effort beyond the roles of student and classroom background attributes. It was therefore important to account for notable student and classroom background attributes. Student background factors were: age (in years); gender (0 = female, 1 = male); socio-economic status (SES), home language background (NESB; 0 = English, 1 = non-English speaking background), and mathematics ability. The SES score was derived from self-rated postcode and/or suburb, using the Australian Bureau of Statistics Index of Relative Socio-Economic Advantage and Disadvantage classification (SEIFA; Australian Bureau of Statistics, 2016), with higher values representative of areas of greater socio-economic advantage.

Mathematics ability was assessed *via* a 10-item mathematics assessment, the High School Mathematics Competency scale (HSMC; Nagy, 2021; and evidence of validation demonstrated in Martin et al., 2020), developed to test the underlying mathematical competencies of students. Assessment items were graduated in difficulty but accessible to all students in years 7–10 without the need for stage-specific subject knowledge. Items were mapped against the New South Wales (NSW) and Australian national curriculums (Australian Curriculum Assessment and Reporting Authority, n.d.; NSW Education Standards Authority, 2019). Example items from this assessment that reflected the curriculum domains of Time, Patterns and Algebra, and Ratios and Rates, were respectively, [Time]: “What time will it be 75 min after 11:15 am? [(A) 11:30 am, (B) 12:30 am, (C) 11:30 pm, and (D) 12:30 pm]”; (Patterns and Algebra): “Find the next number in the pattern: 8, 11, 14, 17, [(A) 20, (B) 21, (C) 22, and (D) 23]”; (Ratio and Rates): “If the ratio of boys: girls in a class is 4:5, what fraction of the class is boys? [(A) 1/4, (B) 1/5, (C) 4/5, and (D) 4/9].” A mathematics ability score was calculated for each student (corresponding to the total number of correct responses out of 10) and then standardized by year group. Three classroom covariates were also included: class size, class-average age, and class-average ability (using the mean mathematics ability score for each classroom).

### Data analyses

Data collected from school students that relates to their learning is typically part of a multilevel structure, with students clustered into classrooms. Within these classrooms, there is generally greater similarity among students than between students of different classrooms, due to factors such as how classroom groupings are chosen (e.g., streaming by ability-level) and unique classroom culture (e.g., due to the unique combination of teacher expectations and classroom climate). Typically, it is statistically invalid to analyze clustered data at a single-level, as it can violate statistical assumptions and give rise to Type 1 errors (Marsh et al., 2008). Furthermore, in measuring and analyzing constructs at either student-level or classroom-level, the interpretation of results may be different and yield different practical implications. It is now well established that accounting for these realities requires multilevel modelling that accommodates the clustering of students within classrooms and distinguishes between student-level effects and classroom-level effects. Indeed, differences in motivation and engagement (specifically, effort, in this study) may be influenced by both individual and classroom factors, and it is therefore appropriate to use a multilevel approach in bringing conceptual and empirical clarity to their association. The central analyses therefore consisted of multilevel confirmatory factor analysis (MCFA) and doubly-latent multilevel structural equation modelling (MSEM).

Analyses were carried out in M*plus* version 8 using maximum likelihood estimation with robust standard errors (MLR; M*plus* RRID:SCR_015578; Muthén and Muthén, 1998–2022), which accounts for non-normality of the sample. Missing data (4%) were handled using the M*plus* full information maximum likelihood (FIML) default. All multilevel modelling included Level 1 (L1; student-level) and Level 2 (L2; classroom-level) variables. To determine model fit, a Comparative Fit Index (CFI) greater than 0.90 and Root Mean Square Error of Approximation (RMSEA) less than 0.08 were used as thresholds for acceptable fit (Hu and Bentler, 1999), and a CFI greater than 0.95 and RMSEA less than 0.05 as thresholds for excellent fit. Prior to conducting multilevel analyses, measurement invariance tests as a function of key sub-groups (e.g., age and gender) were conducted for the effort factors and demonstrated relative invariance across all sub-groups tested. Full details of these tests can be found in Supplementary material in the section titled “Invariance Tests” and in Supplementary Tables S4–S6.

Multilevel descriptive analyses comprised student-level (L1) and classroom-level (L2) scale means, standard deviations, skewness, kurtosis, reliability, and intra-class correlations (ICCs). To test factor structure, two MCFAs were first conducted using the Effort Scale (one involving only first-order effort factors, and the other including a higher-order effort factor). Then, these two MCFAs were re-estimated but with the motivation factors also included. These latter MCFAs enabled a test of fit for models where motivation and effort were represented as distinct factors and an assessment of correlations between motivation and effort. In MCFAs, L1 and L2 parallel latent factor loadings for effort (but not for motivation as these were single-item factors—see Materials) were constrained to be equal (i.e., isomorphism) and L2 residuals were constrained to be greater than zero to ensure a more parsimonious model with greater accuracy in parameter estimation at both levels (e.g., Morin et al., 2014). The hypothesized process model of motivation predicting effort was tested with two doubly-latent MSEMs (one for first-order effort and one for higher-order effort; Figure 1) that included controls for student- and classroom-level background attributes (as predictors of motivation and effort). In the MSEMs, all background covariates were correlated, motivation predictors were correlated, and effort outcomes were correlated.

## Results

### Preliminary descriptive statistics

Means and standard deviations (*SD*s) for effort factors at L1 (student-level) and L2 (classroom-level) are shown in Table 1A. Skewness and kurtosis values are also in Table 1A and are within indicative guidelines for approximately normal distributions (Kim, 2013). Descriptive statistics at L1 and L2 for motivation are displayed in Table 1B, with skewness and kurtosis values also reflecting approximately normal distributions.

**Table 1A:** Multilevel descriptive statistics and CFAs of first-order and higher-order effort.

Variable	Statistics							*M*	*SD*	Skew	Kurtosis	ω	CFA loadings (min., max., mean)	ICC
Level 1 (Student)							
*First-order effort factors*							
Operative effort	5.934	0.955	−1.362	2.755	0.853	0.687, 0.870, 0.808	-
Cognitive effort	5.779	1.051	−1.471	3.225	0.925	0.832, 0.933, 0.896	-
Social-emotional effort	6.243	0.713	−1.102	1.915	0.754	0.660, 0.742, 0.711	-
*Second-order effort factor*							
Higher-order effort	5.985	0.797	−1.093	1.733	0.888	0.698, 0.958, 0.847	-
Level 2 (Classroom)							
*First-order effort factors*							
Operative effort	5.893	0.405	−0.806	0.946	0.985	0.953, 1.000, 0.978	0.087
Cognitive effort	5.739	0.448	−0.637	-0.031	0.999	0.998, 1.000, 0.999	0.104
Social-emotional effort	6.227	0.306	−0.521	-0.281	0.977	0.904, 1.000, 0.966	0.147
*Second-order effort factor*							
Higher-order effort	5.953	0.350	−0.361	-0.445	0.912	0.657, 1.000, 0.870	0.149

ω = reliability (omega total; McNeish, 2018); ICC = Intra Class Correlation; CFA Loadings = Confirmatory factor analysis standardized factor loadings; *M*, *SD*, Skew and Kurtosis are calculated from unit-weighted scale scores of raw items.

**Table 1B:** Multilevel descriptive statistics of motivation items.

Variable	Statistics							*M*	*SD*	Skew	Kurtosis	ω^a^	CFA loading	ICC
Level 1 (Student)							
Self-efficacy (positive motivation)	5.822	1.333	−1.683	2.950	0.771	0.852	-
Valuing (positive motivation)	5.542	1.384	−1.120	1.018	0.770	0.847	-
Mastery orientation (positive motivation)	5.590	1.283	−1.145	1.329	0.806	0.888	-
Anxiety (negative motivation)	5.251	1.754	−0.923	−0.129	0.771	0.759	-
Failure avoidance (negative motivation)	4.715	1.819	−0.474	−0.862	0.766	0.765	-
Uncertain control (negative motivation)	3.012	1.665	0.679	−0.439	0.788	0.821	-
Level 2 (Classroom)							
Self-efficacy (positive motivation)	5.738	0.635	−0.986	0.582	0.777	0.978	0.183
Valuing (positive motivation)	5.507	0.619	−0.578	0.871	0.789	0.971	0.165
Mastery orientation (positive motivation)	5.570	0.445	−0.709	0.595	0.840	0.969	0.106
Anxiety (negative motivation)	5.210	0.493	−0.260	−0.203	0.779	0.936	0.072
Failure avoidance (negative motivation)	4.746	0.605	−0.143	0.428	0.842	0.963	0.098
Uncertain control (negative motivation)	3.120	0.680	0.095	−0.201	0.876	0.980	0.141

ω = reliability (omega total; McNeish, 2018); ICC = Intra Class Correlation; CFA Loadings = Confirmatory factor analysis standardized factor loadings; *M*, *SD*, Skew and Kurtosis are calculated from raw items. ^a^ Motivation items are modelled as error-adjusted scores using established reliability and variance measures from a prior research program (ω and σ^2^ values were derived from data used in: Martin and Marsh, 2005; Marsh et al., 2008).

### Fit and dimensionality of motivation and effort

As described in the Introduction, it is vital to have sound measurement of engagement (by way of effort in this study) in order to effectively explore the distinctiveness of motivation and engagement. Therefore, we first conducted MCFAs to test the hypothesized effort dimensions, operationalized *via* the Effort Scale [see Supplementary material for a summary of single-level (student) CFAs of the Effort Scale]. The first-order effort structure yielded an excellent fit to the data [ $\chi^{2}$ (54) = 177.284, *p* < 0.001, RMSEA = 0.049, CFI = 0.966], as did the higher-order effort structure [ $\chi^{2}$ (56) = 176.868, *p* < 0.001, RMSEA = 0.048, CFI = 0.966]. As Table 1A demonstrates, mean MCFA loadings on the first-order effort factors ranged from 0.71 to 0.90 (L1) and 0.97 to 1.00 (L2), with a grand mean of 0.81 (L1) and 0.98 (L2). The mean MCFA loadings on the higher-order effort factor were 0.85 (L1) and 0.87 (L2). All factor loadings were therefore within an acceptable range (Byrne, 2012). Reliability estimates for first-order effort factors ranged from ω = 0.75 to 0.93 (L1) with a mean of 0.84, and ω = 0.98 to 1.00 (L2) with a mean of 0.99, indicating acceptable internal consistency. Reliability for the higher-order effort factor was ω = 0.89 (L1) and 0.91 (L2) and so also indicated acceptable internal consistency. Table 1A shows intra-class correlations (ICCs) which ranged from 0.09 to 0.15 for first-order effort factors and was 0.15 for the higher-order effort factor. The grand mean ICC (0.12) was above the 10% threshold recommended by Byrne (2012) and provided justification for our multilevel approach in this study.

Having established the dimensionality and measurement properties of effort, we then included motivation in the MCFAs to ascertain its dimensionality and distinctiveness relative to effort. Two models were run^1^ that both yielded excellent fit to the data: one for first-order effort [ $\chi^{2}$ (126) = 296.852, *p* < 0.001, RMSEA = 0.038, CFI = 0.970] and one for higher-order effort [ $\chi^{2}$ (152) = 332.056, *p* < 0.001, RMSEA = 0.035, CFI = 0.969]. Thus, when modelled as separate factors, there is excellent fit, signaling distinct dimensionality between motivation and effort. Table 1B shows motivation factor loadings, determined from a fully-saturated MCFA that only included motivation items, which ranged from 0.76 to 0.89 (L1) with a mean of 0.82 and from 0.94 to 0.98 (L2) with a mean of 0.97. ICCs ranged from 0.07 to 0.18 with a mean of 0.13 indicating that the variance attributable to motivation at the classroom-level was above the recommended threshold (Byrne, 2012) justifying modelling motivation at L1 and L2.

### Multilevel correlations between motivation and effort

The MCFAs involving both motivation and effort also generated latent correlations that were a further means of assessing their distinctiveness. All correlations are summarized in Table 2, with the correlations between the target substantive factors of motivation and effort displayed in bold font for clarity. At both L1 (student-level) and L2 (classroom-level), there were significant positive correlations between all three positive motivation and first- and higher-order effort factors; for operative effort (L1: *r* = 0.50 to 0.53, mean *r* = 0.52, *p* < 0.001; L2: *r* = 0.81 to 0.86, mean *r* = 0.84, *p* < 0.001), cognitive effort (L1: *r* = 0.46 to 0.50, mean *r* = 0.49, *p* < 0.001; L2: *r* = 0.70 to 0.74, mean *r* = 0.72, *p* < 0.001), social–emotional effort (L1: *r* = 0.34 to 0.43, mean *r* = 0.39, *p* < 0.001; L2: *r* = 0.60 to 0.66, mean *r* = 0.64, *p* < 0.001), and higher-order effort (L1: *r* = 0.53 to 0.56, mean *r* = 0.55, *p* < 0.001; L2: *r* = 0.79 to 0.84, mean *r* = 0.82, *p* < 0.001). There were also significant negative correlations between the negative motivation factor of uncertain control and all effort factors: for operative effort (L1: *r* = −0.30, *p* < 0.001; L2: *r* = −0.57, *p* < 0.001), for cognitive effort (L1: *r* = −0.28, *p* < 0.001; L2: *r* = −0.54, *p* < 0.001), for social–emotional effort (L1: *r* = −0.23, *p* < 0.001; L2: *r* = −0.35, *p* < 0.05), and for higher-order effort (L1: *r* = −0.32, *p* < 0.001; L2: *r* = −0.57, *p* < 0.001). There were no significant correlations between the negative motivation factors of anxiety and failure avoidance and any of the effort factors at either level. Taken together, the bivariate associations between motivation and effort demonstrated significant alignments, but at the same time sufficiently sized unshared variance to support their distinctiveness.

**Table 2:** Multilevel correlation matrix within and between motivation and effort factors.

Variables	Operative effort	Cognitive effort	Social–emotional effort	Self-efficacy	Valuing	Mastery orientation	Anxiety	Failure avoidance	Uncertain control
Level 1 (Student)									
*Effort factors*									
Cognitive effort	0.848^***^								
Social–emotional effort	0.611^***^	0.657^***^							
*Motivation factors*									
Self-efficacy (positive)	**0.533** ^***^	**0.503** ^***^	**0.344** ^***^						
Valuing (positive)	**0.533** ^***^	**0.501** ^***^	**0.407** ^***^	0.675^***^					
Mastery orientation (positive)	**0.501** ^***^	**0.461** ^***^	**0.428** ^***^	0.407^***^	0.532^***^				
Anxiety (negative)	**0.079**	**0.019**	**0.071**	−0.158^**^	−0.058	−0.002			
Failure avoidance (negative)	**−0.058**	**−0.055**	**−0.074**	−0.176^***^	−0.191^***^	−0.030	0.537^***^		
Uncertain control (negative)	**−0.301** ^***^	**−0.281** ^***^	**−0.228** ^***^	−0.497^***^	−0.329^***^	−0.181^***^	0.370^***^	0.387^***^	
Level 2 (Classroom)									
*Effort factors*									
Cognitive effort	0.942^***^								
Social–emotional effort	0.721^***^	0.782^***^							
*Motivation factors*									
Self-efficacy (positive)	**0.861** ^***^	**0.744** ^***^	**0.598** ^***^						
Valuing (positive)	**0.851** ^***^	**0.720** ^***^	**0.652** ^***^	0.770^***^					
Mastery orientation (positive)	**0.805** ^***^	**0.699** ^***^	**0.658** ^***^	0.631^***^	0.683^***^				
Anxiety (negative)	**0.149**	**0.085**	**0.217**	0.078	0.044	0.016			
Failure avoidance (negative)	**0.021**	**0.005**	**0.011**	−0.014	−0.043	0.180	0.332^**^		
Uncertain control (negative)	**−0.571** ^***^	**−0.544** ^***^	**−0.354** ^*^	−0.697^***^	−0.527^***^	−0.243	0.062	0.301^*^	
^a^Level 1 Higher order effort				**0.557** ^***^	**0.563** ^***^	**0.529** ^***^	**0.055**	**−0.065**	**−0.316** ^***^
^a^Level 2 Higher order effort				**0.836** ^***^	**0.826** ^***^	**0.792** ^***^	**0.144**	**0.014**	**−0.566** ^***^

Motivation items are modelled as error-adjusted scores; All correlations taken from the first-order CFA model with the exception of ^a^higher-order effort correlations which are taken from the higher-order CFA model; values in bold highlight correlations between the substantive factors; ^*^p < 0.05. ^**^p < 0.01. ^***^p < 0.001.

### Multilevel structural equation modelling of motivation predicting effort

**Figure 2:**
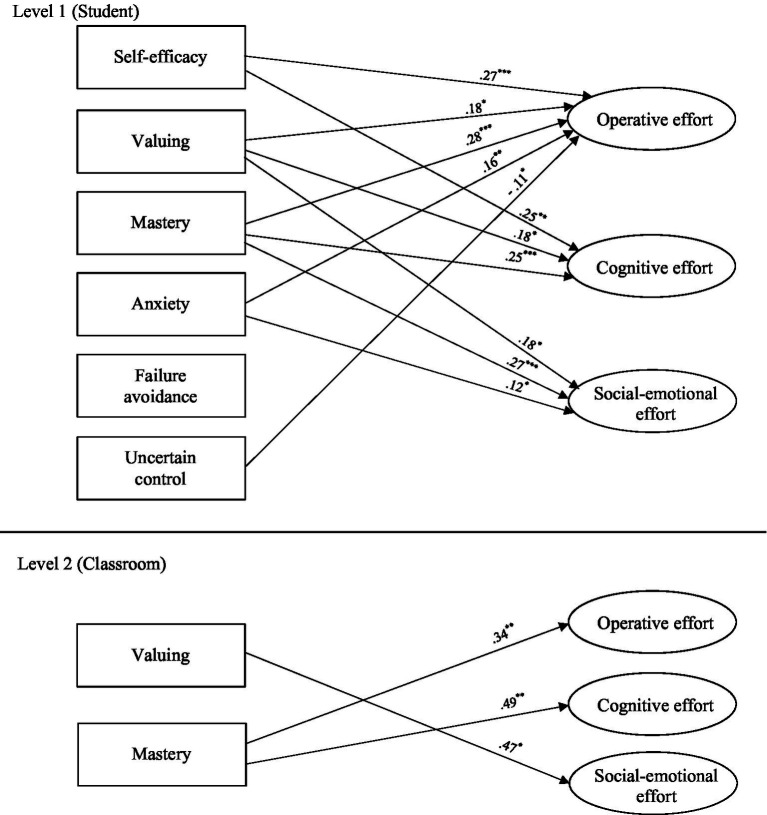
Significant substantive paths in central multilevel analysis—First order effort factors (MSEM_1_). Only significant paths are shown and are labeled with standardized betas (β); ^*^p < 0.05. ^**^p < 0.01. ^***^p < 0.001; All paths controlled for variance attributed to covariates (Level 1: age, gender, social-economic status, non-English speaking background, mathematics ability; Level 2: class-average ability, class size and class-average age). See Table 3 for all covariate associations and all non-significant paths.

**Figure 3:**
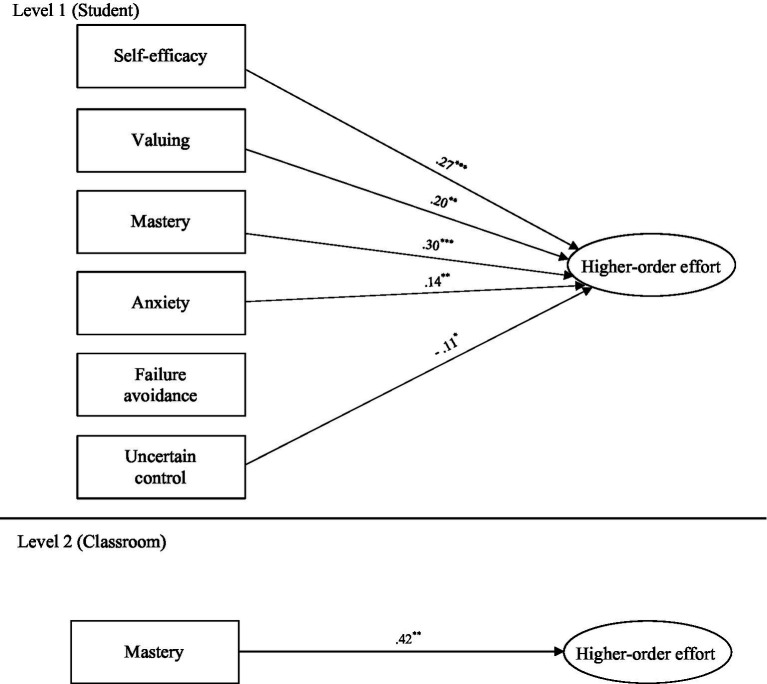
Significant substantive paths in central multilevel analysis—Higher Order Effort Factor (MSEM_2_). Only significant paths are shown and are labeled with standardized betas (β); ^*^p < 0.05. ^**^p < 0.01. ^***^p < 0.001; All paths controlled for variance attributed to covariates (Level 1: age, gender, social-economic status, non-English speaking background, and mathematics ability; Level 2: class-average ability, class size, and class-average age). See Table 3 for all covariate associations and all non-significant paths.

The multilevel process model (Figure 1) of motivation predicting effort was then tested using doubly-latent MSEM. Two MSEMs were conducted, the first (MSEM_1_) examined motivation predicting first-order effort factors, and the second (MSEM_2_) investigated motivation predicting higher-order effort. To appropriately ascertain the unique associations between motivation and effort (beyond student and classroom background attributes), the MSEMs included controls for a range of student covariates (age, gender, SES, NESB, and mathematics ability) and classroom-level attributes (class-average ability, class size, and class-average age)—with these covariates predicting motivation and effort in the MSEMs. Both models yielded an excellent fit to the data [first-order effort model MSEM_1_: $\chi^{2}$ (174) = 365.398, *p* < 0.001, RMSEA = 0.034, CFI = 0.971; higher-order effort model MSEM_2_: $\chi^{2}$ (216) = 458.662, *p* < 0.001, RMSEA = 0.034, CFI = 0.964]. In the summary of substantive findings described here, only significant L1 and L2 standardized paths (β) between the substantive factors and notable results involving covariates are presented (and these are shown in Figures 2 and 3 for the first-order effort and higher-order effort models respectively). All significant and non-significant standardized substantive and covariate paths are reported in Table 3.^2^

**Table 3:** Multilevel structural equation process model: Standardized beta coefficients.

Variables	Outcomes (MSEM_1_ using first-order effort factors)									MSEM_2_
	Self-efficacy	Valuing	Mastery orientation	Anxiety	Failure avoidance	Uncertain control	Operative effort	Cognitive effort	Social–emotional effort	Higher-order effort
Level 1 (Student)										
*L1 Covariates*										
SES	0.056	0.035	0.007	0.063	0.012	0.012	0.023	−0.018	−0.083^*^	−0.010
Age	0.022	0.034	0.028	−0.032	−0.025	−0.014	−0.010	−0.018	−0.015	−0.016
Gender (male)	0.158^***^	0.101^**^	0.041	−0.162^***^	−0.034	−0.086^**^	−0.099^*^	−0.035	−0.236^***^	−0.087^*^
NESB	−0.045	0.052	−0.002	−0.025	0.061	0.070	−0.046	−0.022	−0.035	−0.036
Mathematics ability	0.213^***^	0.160^***^	0.044	−0.070	−0.123^**^	−0.142^**^	0.056	0.052	0.059	0.061^*^
*L1 Motivation factors*										
Self-efficacy (positive)							0.270^***^	0.252^**^	0.099	0.272^***^
Valuing (positive)							0.179^*^	0.177^*^	0.175^*^	0.198^**^
Mastery orientation (positive)							0.278^***^	0.248^***^	0.274^***^	0.295^***^
Anxiety (negative)							0.162^**^	0.087	0.121^*^	0.138^**^
Failure avoidance (negative)							−0.005	0.025	−0.037	0.005
Uncertain control (negative)							−0.114^*^	−0.089	−0.107	−0.113^*^
Level 2 (Classroom)										
*L2 Covariates*										
Class-average ability	0.368^**^	0.437^**^	−0.127	−0.097	−0.550^***^	−0.629^***^	0.132	0.337	0.351^*^	0.238
Class size	0.298^*^	−0.008	0.379^*^	0.292	0.407^*^	−0.142	−0.093	−0.259	−0.269	−0.171
Class-average age	−0.153	−0.361^***^	−0.287^**^	0.204	−0.036	−0.101	−0.178	−0.102	0.113	−0.135
*L2 Motivation factors*										
Self-efficacy (positive)							0.319	0.236	0.177	0.292
Valuing (positive)							0.193	0.000	0.465^*^	0.149
Mastery orientation (positive)							0.340^**^	0.490^**^	0.390	0.423^**^
Anxiety (negative)							0.101	0.082	0.015	0.101
Failure avoidance (negative)							0.014	0.061	0.006	0.027
Uncertain control (negative)							−0.158	−0.197	0.112	−0.148

Motivation items modelled as error-adjusted scores. SES, Social-economic status indicator (positive is higher SES); NESB, Non-English speaking background; ^*^p < 0.05. ^**^p < 0.01. ^***^p < 0.001.

At L1, student-level self-efficacy significantly positively predicted student-level operative effort (β = 0.27, *p* < 0.001), cognitive effort (β = 0.25, *p* < 0.01), and higher-order effort (β = 0.27, *p* < 0.001). Student-level valuing significantly positively predicted student-level operative effort (β = 0.18, *p* < 0.05), cognitive effort (β = 0.18, *p* < 0.05), social–emotional effort (β = 0.18, *p* < 0.05), and higher-order effort (β = 0.20, *p* < 0.01). Student-level mastery orientation significantly positively predicted student-level operative effort (β = 0.28, *p* < 0.001), cognitive effort (β = 0.25, *p* < 0.001), social–emotional effort (β = 0.27, *p* < 0.001), and higher-order effort (β = 0.30, *p* < 0.001). Examining the negative motivation factors at L1, student-level uncertain control significantly negatively predicted student-level operative effort (β = −0.11, *p* < 0.05) and higher-order effort (β = −0.11, *p* < 0.05). Interestingly, student-level anxiety significantly positively predicted student-level operative effort (β = 0.16, *p* < 0.01), social–emotional effort (β = 0.12, *p* < 0.05), and higher-order effort (β = 0.14, *p* < 0.01). At L2, the significant paths found between the classroom-level motivation and effort factors were in relation to valuing, which positively predicted social–emotional effort (β = 0.47, *p* < 0.05), and mastery orientation, which positively predicted operative effort (β = 0.34, *p* < 0.01), cognitive effort (β = 0.49, *p* < 0.01), and higher-order effort (β = 0.42, *p* < 0.01).

Although not the substantive focus of the study, for completeness we report here noteworthy patterns of covariate associations where a given L1 (student-level) or L2 (classroom-level) covariate significantly predicted both motivation and effort (see Table 3 for all covariate associations). At L1, gender significantly predicted student-level motivation and effort. Specifically, being male was positively associated with self-efficacy (β = 0.16, *p* < 0.001), and valuing (β = 0.10, *p* < 0.01), and negatively associated with anxiety (β = −0.16, *p* < 0.001), uncertain control (β = −0.09, *p* < 0.01), operative effort (β = −0.10, *p* < 0.05), social–emotional effort (β = −0.24, *p* < 0.001), and higher-order effort (β = −0.09, *p* < 0.05). Student-level mathematics ability significantly positively predicted self-efficacy (β = 0.21, *p* < 0.001), valuing (β = 0.16, *p* < 0.001), and higher-order effort (β = 0.06, *p* < 0.05), and negatively predicted failure avoidance (β = −0.12, *p* < 0.01), and uncertain control (β = −0.14, *p* < 0.01). At L2, class-average ability significantly positively predicted self-efficacy (β = 0.37, *p* < 0.01), valuing (β = 0.44, *p* < 0.01), and social–emotional effort (β = 0.35, *p* < 0.05), and negatively predicted failure avoidance (β = −0.55, *p* < 0.001), and uncertain control (β = −0.63, *p* < 0.001).

## Discussion

The present study sought to bring new insights to the alignment and distinctiveness of motivation and engagement (operationalized as effort). Beginning with a conceptual review to clarify definitional parameters of both motivation and engagement (with specific focus on the relatively neglected construct of effort), we tested a hypothesized multidimensional effort structure and then its empirical juxtaposition with a well-established motivation framework. Multilevel (student- and classroom-level) findings supported the reliability and validity of multidimensional effort (by way of the Effort Scale) and the distinctiveness of effort from motivation by way of multidimensional model fit and latent bivariate multilevel correlations. Then, MSEM explored the “classic” motivation → engagement (effort) process. This revealed significant associations between student- and classroom-level motivation and student- and classroom-level effort—as well as some noteworthy patterns of covariates predicting both motivation and effort at student- and classroom-levels. These findings and their implications for motivation and engagement theory, research, and practice are now discussed.

### Findings of note

This study has not only reinforced well-established understanding of motivation and engagement as two inter-related constructs (Martin, 2009; Martin et al., 2017), it has also shed new light on some of the precise ways in which individual motivation factors interplay with specific multidimensional engagement factors. MCFA findings showed multidimensional motivation and multidimensional effort to have distinct factor structures, with significant and theoretically plausible bivariate correlations between first-order motivation and first- and higher-order effort factors. MSEM further supported this *via* unique predictive associations between first-order motivation, and first- and higher-order effort factors. In this study we were especially interested in the extent to which motivation is associated with effort in theoretically plausible ways (convergent validity) and also the extent to which there remained sufficient unshared variance to indicate their distinctiveness (discriminant validity). Our findings garner strong evidence for both convergent (significant associations) and discriminant validity (noteworthy unshared variance) between motivation and effort.

The MSEM provided a particularly nuanced insight into how multidimensional motivation and effort are aligned and distinct, bringing greater psychometric clarity to developments in theorizing, and affording a better understanding of the distinctiveness and interface of motivation and engagement (by way of our novel effort framework). Positive motivation factors were found to overwhelmingly predict effort at the student-level. Specifically (after controlling for student-level background attributes—discussed below), mastery orientation and valuing uniquely predicted all three effort factors (operative, cognitive, and social–emotional), and self-efficacy predicted both operative and cognitive effort. All three positive motivation factors predicted higher-order effort. In explaining the salient role of mastery orientation, it is worth noting central tenets of goal theory (Elliot, 2005) that posits effort as a means by which students’ mastery orientation is operationalized. Indeed, classroom-level mastery orientation also predicted classroom-level effort, which is in line with the role of classroom motivational climates in classroom-level engagement under goal theory (Ames, 1992; Wentzel, 2012; Wentzel et al., 2017). There are thus strong theoretical roots underpinning the role of mastery orientation in predicting effort at both student- and classroom-levels.

Valuing was also predictive of all three effort dimensions at the student-level and of social–emotional effort at the classroom-level. Thus, when an individual student believes in the importance and relevance of their academic work to learning, they are more likely to try harder in their application to that learning. This finding aligns with major psycho-educational perspectives—particularly, expectancy-value theory—contending that “students’ subjective task values predict both intentions and actual decisions to persist at different activities” (Wigfield and Cambria, 2010, p. 21). In addition, Wigfield and Cambria (2010) highlight that students’ values are socio-culturally situated which may well explain why, at the classroom-level, valuing predicted social–emotional effort. Indeed, Eccles and Wigfield (2020) recently updated their conceptual framework to “situated expectancy-value theory” to reflect the situated nature of motivation and motivational processes. In the case of our study, classrooms comprising students who view academic tasks as more important (higher classroom-average valuing) seemed to be contexts conducive to greater class-average extension of interpersonal respect and self-control (higher classroom-average social–emotional effort). It is interesting that class-average valuing did not significantly predict either operative or cognitive effort at the classroom-level. The reason for this is not clear, but there may be something about classroom-level valuing that lends to classroom-level interpersonal prosocial behavior (in the form of social–emotional effort) but not classroom-level intrapersonal behavior (in the forms of operative and cognitive effort) that requires further investigation (see Warrington and Younger, 2011 for an example of related research identifying the role of peer group inclusion and exclusion in school).

It was also interesting to note that student-level self-efficacy, although predictive of students’ operative and cognitive effort, did not significantly predict students’ social–emotional effort. This suggests that the belief and confidence that students have about their own ability is reflected more towards the effort they invest in their own personal application and cognition rather than towards their inter-personal self-regulation and demonstration of respect for others. This confirms that self-efficacy as a motivational driver is associated more with what Bandura (2001) described as direct personal agency, than to other-oriented agency.

Another result warranting further consideration is that of student-level anxiety (a negative motivation factor) positively predicting operative, social–emotional, and higher-order effort. One could be forgiven for expecting that anxious students would be more avoidant or debilitated in their effort/engagement (Yang et al., 2021; Quintero et al., 2022). However, our results indicate that anxiety is a potentially arousing (rather than debilitating) factor—in line with classic cognitive appraisal theories where task demands can be perceived as challenges more than threats (e.g., Lazarus and Folkman, 1984). Of course, another interpretation is that students responded to their anxiety with greater effort so they could avoid the poor performance they are anxious about (see Covington, 2000; Martin et al., 2003). However, failure avoidance did not predict effort at either student-or classroom-levels and so we believe we can discount this possibility.

Notwithstanding mastery orientation and valuing, our findings showed that the link between motivation and effort is predominantly manifested between students rather than between classrooms. This is consistent with findings of other studies demonstrating that the majority of variance in motivation and engagement occurs at the student-level (e.g., Martin and Marsh, 2005). At the same time, however, there was a more consistent pattern of classroom-level background attributes that predicted motivation and effort—and in fact, more so for motivation than for effort. Specifically, our findings indicated that: classroom-average ability was associated with higher positive motivation and lower negative motivation, in line with prior motivation research (see Elliot, 2005); classroom-average age was negatively associated with positive motivation factors, consistent with well-documented developmental declines in motivation (e.g., Jacobs et al., 2002; Gottfried et al., 2007); and, class size positively predicted positive motivation, but also positively predicted failure avoidance, potentially reflecting the somewhat equivocal results in class size research over the past five decades (e.g., see Glass and Smith, 1979; Blatchford, 2011).

Turning to the student-level background attributes, gender was the only factor predicting both motivation and effort. Interestingly, despite having higher positive motivation and lower negative motivation, boys were also significantly less likely than girls to invest this motivation in academic effort. Indeed, other research has also suggested that boys are higher than girls in some aspects of motivation (perceived competence) but lower in effort (Wilkie, 2019). Why this is the case requires further investigation, but we suspect answers may lie in gender-specific constructions of effort. For example, research has shown that being seen to put effort into academic work may not fit with culturally prescribed representations of masculinity (Connell and Messerschmidt, 2005) or what is considered “cool” for boys to do (Martino, 1999, 2000; Jackson and Dempster, 2009). Perhaps in some support of this, our results showed that it was the more visible and observable aspects of effort (operative and social–emotional) where boys scored lower, not the internal (cognitive) aspect of effort. Taken together, these findings have highlighted some of the student and context background attributes that are important to include in research seeking to better understand the salient alignments and distinctions between motivation and engagement.

### Implications for theory and research

In line with the call for new expositions of engagement to advance the field (Reschly and Christenson 2012), we sought to bring greater lucidity to the motivation and engagement space through a purposeful focus on effort (a specific active form of engagement) and how it relates to multidimensional motivation. In this way, our findings build on recent developments in concepts and theory, helping to further understand the distinctiveness of motivation and engagement, the interface between them, and the interplay between their individual dimensions. For example, it supported theorized distinctions between internal and external aspects of motivation and engagement (Martin, 2012, 2022) in that there was clear measurement and correlational distinction between the study’s motivation and effort factors. As noted above, findings also shed light on what aspects of major motivational theories [e.g., goal theory regarding mastery orientation, Elliot, 2005; (situated) expectancy-value theory regarding valuing, Eccles and Wigfield, 2020] are associated with distinct aspects of engagement. By introducing a novel engagement framework by way of multidimensional effort, our findings extend claims made by these theories with respect to motivation and its academic effects.

The study also offers measurement yields. To capture our hypothesized multidimensional effort framework, we developed and established evidence for the validity of a novel instrument—the Effort Scale—that assessed three distinct aspects of effort (operative, cognitive, and social–emotional) in line with its overarching umbrella construct, tripartite engagement (Fredricks et al., 2004). This study therefore offers future researchers a feasible new method of studying effort (as a pertinent example of active classroom engagement). In addition to the Effort Scale, in Supplementary material, we also established evidence for the validity of a parallel three-item version (the Effort Scale—Short) that may be useful in research where longer forms are not feasible (e.g., in real-time research, intensive longitudinal work, etc. see Gogol et al., 2014; Martin et al., 2020).

### Implications for practice

The dominant pattern of findings suggests the importance of targeting self-efficacy, valuing, and mastery orientation—as these were the main predictors of effort. Martin (2007) gives some practical examples to develop each of these facets; for instance, the restructuring of learning to maximize opportunities for success may boost students’ *self-efficacy*, as might enhancing students’ beliefs about themselves and their academic capabilities, and developing their skills in effective goal-setting to boost competence. Providing students with relevance and meaning in their learning is one way of improving *valuing* (Martin, 2007), which is further enhanced by teachers modeling positive attitudes in valuing what they teach (Eccles and Wigfield, 2002). *Mastery orientation* can be enhanced by focusing students on the task at hand more than on the assessment grade associated with it (Martin, 2007), and also on students’ own personal learning and progress more than how they compare and compete with other students (Martin and Elliot, 2016).

Alongside motivational intervention as a means of enhancing effort, it is also important to boost effort directly. This is where the multidimensional perspective on effort is especially useful, as it enables targeted and specific educational action. *Operative effort* may be supported by teachers encouraging students to complete schoolwork by the given deadline, emphasizing the importance of students’ active investment of time and energy in the completion and quality of their academic work. Teachers who regularly check students’ work are best placed to assess their operative effort, and in doing so, actively encourage such effort by commending students for trying hard where applicable. Teachers can also suitably acknowledge students’ proactive academic output that is additional to the minimum specified task requirements, encouraging students to engage in supplementary practice, where appropriate, to cement understanding and techniques.

*Cognitive effort* may be targeted by encouraging students to develop ‘active listening’ and attentional skills (e.g., presenting positive indications of concentration and focus during instruction, such as eye-contact), commending students for their focus, and reflective thinking in their comments and academic work, where appropriate. Another strategy that can be adopted is for teachers to explicitly promote cerebral challenge (or “brain burn”; for specific examples appropriate to the mathematics classroom, such as the metaphoric “brain gym” see Nagy, 2013). Teachers can promote cognitive effort by allowing students sufficient processing time before eliciting responses to questions that arise in class discussions, affording students more opportunity to think about questions, and formulate proactive contributions to class discussions to clarify their developing schema. Students should be encouraged, where appropriate, to extend their learning by engaging in mentally challenging tasks, and to use cognitive strategies such as visualization and self-talk. A further strategy to improve cognitive effort is for students to increase the duration and frequency of their quiet task-focus time, in class and at home, including turning off mobile phones and social media notifications.

Teachers can enhance students’ *social–emotional effort* by developing clear classroom expectations of mutual support and respect and being explicit about the behaviors they want sustained, such as interest in others’ classroom contributions, support for others’ participation, management of impulsivity, proactive self-regulation, and contribution to positive classroom culture. At the same time, teachers might seek to eliminate behaviors that are not acceptable, such as derision of others’ contributions and achievements, shouting out, talking over others, not taking turns, and so on—so that they foster a social–emotional classroom that is a safe environment in which to explore and test ideas and critical thinking.

Not only does the study suggest direction on the motivation and effort factors to target, it also provides direction on the students and classrooms for whom boosting motivation and effort is particularly important. For example, the findings suggest the need to target boys’ operative and social–emotional effort. It is also evident that girls may need help to reduce anxiety and uncertain control, alongside support to boost their self-efficacy and valuing. The study also suggests improving social–emotional effort among students in low ability classrooms and reducing failure avoidance in larger classes. When considering these students and classrooms for applied focus, it is worth remembering that the present study was conducted in the mathematics domain and it is known that this is an area where, for example, there are gender differences in motivation (Meece et al., 2006; Watt et al., 2012) and also motivation and engagement differences as a function of ability (Wang and Eccles, 2013). Indeed, as discussed in Limitations below, the extent to which this study’s findings and practical advice apply to other subject domains remains to be investigated.

### Limitations and future directions

There are some limitations to consider when interpreting our results and which provide potential direction for future research. First, the correlational approach in this study cannot be interpreted as supporting causal conclusions. Experimental and longitudinal designs are required to establish the causal ordering of motivation and engagement (effort) implied in our research. Second, as noted above, our study targeted motivation and engagement in mathematics. Further research is needed to verify the extent to which results are replicated in other subject areas. Third, data were collected *via* self-reports, reflecting students’ perceptions of their motivation and effort. Recent research (e.g., Collie and Martin, 2017) has highlighted the importance of garnering perspectives from multiple informants (e.g., in this case, the students and their teachers) in order to obtain a more comprehensive understanding of a target construct like effort, that comprises both internal and external facets. Fourth, although we established illuminating associations between motivation and effort, an extension of the present study might investigate a fuller process, such as including achievement in our hypothesized process as a consequence of effort. Fifth, motivation was assessed using single-item indicators, modelled as error-adjusted scores using established reliability and variance measures from a prior research program. Further studies might consider using multiple-item latent motivation measures to ensure greater measurement accuracy. Sixth, our analyses were based on variable-centered techniques (MCFA, MSEM) which highlight associations between variables at a whole-sample level, but may mask important findings that are pertinent to subpopulations within the sample. Person-centered techniques such as latent profile analysis may identify effort profiles among particular subpopulations of students that are not evident in variable-centered approaches. Seventh, data collection took place at the end of 2020, in the first year of the COVID-19 pandemic. There was some minor disruption to learning earlier in the year, but Australian schools had returned to face-to-face learning for a period of 6 months prior to data collection, and as such we do not expect this to have significantly impacted results. Finally, our sample comprised Year 7–10 Australian high-school students from independent schools. It is important to expand the age-range, national context, and type of school sampled in future studies to establish the generality of the present findings.

## Conclusion

This study sought to shed further light on the unique and shared variance between motivation and engagement (by way of effort). The findings have provided several avenues of focus for subsequent motivation research and theorizing. They have also established evidence for the validity of a novel engagement framework (multidimensional effort) that may support future measurement and practice in academic engagement. In so doing, the research presented here offers greater clarity and coherence for educational researchers and practitioners in their approaches to optimizing students’ academic development.

## Data availability statement

The datasets presented in this article are not readily available because consent from participants to share the dataset is not available. Summative data are available and can be requested. Requests to access the datasets should be directed to AM; andrew.martin@unsw.edu.au.

## Ethics statement

The studies involving human participants were reviewed and approved by UNSW Human Ethics Committee (Approval #HC200273). Written informed consent to participate in this study was provided by the participants’ legal guardian/next of kin.

## Author contributions

RN shared in the development of the research and materials design and led data analysis and report writing. AM and RC shared in the development of the research and materials design and assisted with data analysis and report writing. All authors contributed to the article and approved the submitted version.

## Conflict of interest

One of the measures (the MES) in the study was developed by the second author, AM, and is a published instrument attracting a small fee, of which a part is put towards its ongoing development and administration, and part of which is also donated to UNICEF. However, for this study, there was no fee involved for its use.

The remaining authors declare that the research was conducted in the absence of any commercial or financial relationships that could be construed as a potential conflict of interest

The handling editor FG declared a past co-authorship with the authors AM and RC.

## Publisher’s note

All claims expressed in this article are solely those of the authors and do not necessarily represent those of their affiliated organizations, or those of the publisher, the editors and the reviewers. Any product that may be evaluated in this article, or claim that may be made by its manufacturer, is not guaranteed or endorsed by the publisher.

## Footnotes

^1^For completeness we also tested a model where the six motivation factors and three first-order effort factors loaded onto a single higher-order factor (thus, a model where motivation and effort were not differentiated as separate constructs). This yielded a significantly poorer fit to the data relative to the excellent fit of the MCFAs separating motivation and effort as distinct constructs: ( $\chi^{2}$ [188] = 775.512, *p* < 0.001, RMSEA = 0.057, CFI = 0.897).

^2^Full results of the higher-order effort model (MSEM_2_) can be seen in Supplementary Material Table S7.
