# Corrigendum: Disentangling motivation and engagement: Exploring the role of effort in promoting greater conceptual and methodological clarity

**DOI:** 10.3389/fpsyg.2024.1412096

**Published:** 2024-05-09

**Authors:** Robin P. Nagy, Andrew J. Martin, Rebecca J. Collie

**Affiliations:** School of Education, University of New South Wales, Sydney, NSW, Australia

**Keywords:** motivation, effort, engagement, academic development, validity, multilevel

In the published article, there was an error in **Supplementary Table S3**. The second item (Cognitive Effort) should say “I focus well, stay on task, and pay attention” but it mistakenly said “I focus well, am on task, and get involved in activities”.

The authors apologize for this error and state that this does not change the scientific conclusions of the article in any way. The original Supplementary material has been updated.

