# Supplementary material for: Disentangling motivation and engagement: Exploring the role of effort in promoting greater conceptual and methodological clarity
Source: Front Psychol. 2022 Dec 13;13:1045717. doi: 10.3389/fpsyg.2022.1045717 (PMC9793434; doi:10.3389/fpsyg.2022.1045717)
Supplement: Supplementary file 1 [file Data_Sheet_1.pdf]

# SUPPLEMENTARY MATERIAL

https://www.frontiersin.org/articles/10.3389/fpsyg.2022.1045717/full#supplementary-material

for

## Disentangling Motivation and Engagement: Exploring the Role of Effort in Promoting Greater Conceptual and Methodological Clarity

(2022) *Frontiers in Psychology* 13:1045717. DOI: 10.3389/fpsyg.2022.1045717

Robin P. Nagy, Andrew J. Martin*, and Rebecca J. Collie

School of Education, University of New South Wales, Sydney, NSW, Australia

*** Correspondence**
Requests for further information about this investigation can be made to Professor Andrew J. Martin, School of Education, University of New South Wales, NSW 2052, AUSTRALIA. E-Mail: andrew.martin@unsw.edu.au.

### Summary of Single-level (Student) CFAs of the Effort Scale

The nine Effort Scale items (three items in each dimension of operative, cognitive and social-emotional effort) are displayed in Table S1. All items were rated using a 7-point Likert scale (1 = *strongly disagree* to 7 = *strongly agree*). We conducted two preliminary single-level CFAs at L1 (student-level) to provide a descriptive overview of the underlying measurement properties of the Effort Scale. A summary of the descriptive statistics for the Effort Scale is displayed in Table S2, including scale means, standard deviations, skewness, kurtosis, reliability, and range and means of factor loadings. Skewness and kurtosis of all measures were within indicative guidelines for approximately normal distributions (Kim, 2013). The first CFA involved first-order effort factors only, and the second included a higher-order effort factor. The first-order effort structure yielded an excellent fit to the data ($\chi^2$[24] = 105.191, $p < .001$, RMSEA = .060, CFI = .967), as did the higher-order effort structure ($\chi^2$[24] = 105.190, RMSEA = .060, CFI = .967). As can be seen from Table S2, factor loadings on the three first-order effort dimensions ranged from .69 to .94 with a grand mean of .82. CFA loadings on the higher-order effort factor ranged from .70 to .97 with a mean of .85. All factor loadings were therefore within an acceptable range (Byrne, 2012). The reliability estimates for the first-order effort factors (omega total; McNeish, 2018) ranged from ω = .78 to .93 with a mean of .86, and for the higher-order effort factor was ω = .89, indicating acceptable internal consistency of the Effort Scale.

### Auxiliary Analysis of the Effort Scale – Short (ES-S)

Further analyses were conducted into a shorter 3-item version of the Effort Scale, the Effort Scale - Short (ES-S), detailed in Table S3, with one item assessing each dimension of effort. Operative effort was measured via the item "In mathematics, I try to the best of my ability on schoolwork (in class or at home etc.)"; cognitive effort was measured via the item "In mathematics, I focus well, stay on task, and pay attention", and social-emotional effort was measured via the item "In mathematics, I show self-control and respect to others (e.g., I wait my turn, don't interrupt etc.)". Items were rated

*Copyright © 2022 Nagy, Martin and Collie. This is an open-access article distributed under the terms of the Creative Commons Attribution License (CC BY). The use, distribution or reproduction in other forums is permitted, provided the original authors and copyright owners are credited and that the original publication in this journal is cited, in accordance with accepted academic practice. No use, distribution or reproduction is permitted which does not comply with these terms.*

using a 7-point Likert scale (1 = *strongly disagree* to 7 = *strongly agree*). Parallel analyses to the full effort scale (MCFA, multilevel correlations, and MSEM) were carried out to establish validity and reliability for the scale. A preliminary fully-saturated single-level CFA was also carried out at L1 (student-level) to provide a descriptive overview of the underlying measurement properties of the ES-S. A summary of these descriptive statistics for the ES-S is displayed in Table S2, alongside the equivalent measures from the full Effort Scale. Skewness and kurtosis of all measures were within indicative guidelines for approximately normal distributions (Kim, 2013).

## Invariance Tests

Prior to conducting the multilevel (student- and classroom-level) analyses, the underlying measurement properties of the data were first inspected at the student-level—as a preliminary basis upon which to proceed to multilevel models. In addition to multi-factor confirmatory factor analysis (CFA), this included tests of measurement invariance for effort as a function of key sub-groups (i.e., gender, age, NESB, and SES). Multigroup tests of metric invariance were conducted for the effort factors, with CFA loadings restricted to be the same across covariate groupings. Tests were carried out separately using the 3-factor first-order effort model, the 4-factor higher-order effort model, and the Effort Scale – Short 1-factor model. These invariance tests examined incremental change in CFI and RMSEA fit indices (with change in RMSEA significant if > .015, and change in CFI significant if < -.01; Cheung & Rensvold, 2002). The full results of these tests are detailed in Tables S4 (first-order 3-factor), S5 (higher-order 4-factor), and S6 (Effort Scale – Short 1-factor), and demonstrate relative invariance across all sub-groups tested. On this basis, the data were pooled and whole-sample analyses were conducted in all subsequent multilevel modelling.

### Multilevel Descriptive Statistics and Model Fit of Short-Scale Effort

L1 (student-level) and L2 (classroom-level) means and standard deviations for motivation factors, and for the short-scale effort factor, are shown in Table S8. Skewness and kurtosis values are also in Table S8 and within indicative guidelines for approximately normal distributions (Kim, 2013).

An MCFA was conducted with motivation factors and the short-scale effort factor. The MCFA yielded an excellent fit to the data ($\chi^2[26] = 24.686$, $p < .001$, RMSEA = .000, CFI = 1.000). As Table S8 demonstrates, MCFA loadings on the short-scale effort factor ranged from .57 to .81 (L1) and .81 to 1.00 (L2), with a mean of .73 (L1) and .92 (L2). All factor loadings were therefore within an acceptable range (Byrne, 2012). Reliability estimates for short-scale effort factor were $\omega$ = .78 (L1), and $\omega$ = .95 (L2), indicating acceptable internal consistency. Table S8 also shows the intra-class correlation (ICC) for short-scale effort which was .24 and thus above the 10% threshold recommended by Byrne (2012) providing justification for multilevel analyses.

### Multilevel Correlations between Motivation and Short-Scale Effort

The MCFAs involving both motivation and effort also generated latent correlations that are summarized in Table S9. Correlations between short-scale effort and motivation were similar to respective correlations for higher-order effort in the full Effort Scale (reported in Table 2 of the main study). Specifically, there were significant correlations at L1 with self-efficacy ($r = .59$, $p < .001$), valuing ($r = .60$, $p < .001$), mastery orientation ($r = .58$, $p < .001$), and uncertain control ($r = -.27$, $p < .001$). At L2, the correlations between short-scale effort and motivation were also similar to respective correlations for higher-order effort in the full Effort Scale. Specifically, L2 self-efficacy ($r = .78$, $p < .001$), valuing ($r = .79$, $p < .001$), mastery orientation ($r = .78$, $p < .001$), and uncertain control ($r = -.56$, $p < .001$). When comparing the similarity of these short-scale effort correlations with correlations for the full scale in the main study (see Table 2 of the main study), we conclude there is evidence for the validity of the Effort Scale - Short as a brief proxy for the full Effort Scale.

*Copyright © 2022 Nagy, Martin and Collie. This is an open-access article distributed under the terms of the Creative Commons Attribution License (CC BY). The use, distribution or reproduction in other forums is permitted, provided the original authors and copyright owners are credited and that the original publication in this journal is cited, in accordance with accepted academic practice. No use, distribution or reproduction is permitted which does not comply with these terms.*

**Multilevel Structural Equation Modelling (MSEM) of Motivation Predicting Short-Scale Effort**

The multilevel process model (Figure 1 of main study) of motivation predicting short-scale effort was then tested using a doubly-latent MSEM. The MSEM included controls for a range of student covariates (age, gender, SES, NESB, mathematics ability) and classroom-level attributes (class-average ability, class size, class-average age)—with these covariates predicting motivation and effort in the MSEM. The model yielded an excellent fit to the data ($\chi^2[216] = 458.662$, $p < .001$, RMSEA = .034, CFI = .964). All significant and non-significant standardized substantive and covariate paths are reported in Table S10.

The significant L1 and L2 standardized paths (β) between the motivation and effort factors (using short-scale effort) were similar to those in the main study (using higher-order effort from the full Effort Scale; see Table 3 of the main study). At L1, student-level self-efficacy significantly positively predicted effort (β = .31, $p < .001$); valuing positively predicted effort (β = .22, $p < .05$) and mastery orientation positively predicted effort (β = .34, $p < .001$). Unlike the full Effort Scale in the main study, none of the negative motivation factors at L1 significantly predicted short-scale effort. However, in line with the full effort scale in the main study, at L2, mastery orientation, positively predicted short-scale effort (β = .54, $p < .001$).

Similar L1 covariate predictions were found for short-scale effort as for full-scale effort in the main study, but there were no significant L2 covariate predictions with short-scale effort. Full details of all substantive and covariate paths are displayed in Table S10.

In conclusion, the Effort Scale - Short measure has proved to be substantially similar to the full Effort Scale in terms of descriptive statistics, correlations and associations with motivation factors and covariates, although not as finely nuanced in terms of its reference frame of overall effort rather than more specific first-order and higher-order effort factors. Nevertheless, we anticipate that it will prove to be a useful measure in studies that wish to include academic effort succinctly as part of a range of different variables.

*Copyright © 2022 Nagy, Martin and Collie. This is an open-access article distributed under the terms of the Creative Commons Attribution License (CC BY). The use, distribution or reproduction in other forums is permitted, provided the original authors and copyright owners are credited and that the original publication in this journal is cited, in accordance with accepted academic practice. No use, distribution or reproduction is permitted which does not comply with these terms.*

## References

Byrne, B. M. (2012). *Structural equation modeling with Mplus: Basic concepts, applications, and programming*. Routledge. https://doi.org/10.4324/9780203807644

Cheung, G. W., & Rensvold, R. B. (2002). Evaluating goodness-of-fit indexes for testing measurement invariance. *Structural Equation Modeling*, *9*(2), 233–255. https://doi.org/10.1207/S15328007SEM0902_5

Kim, H.-Y. (2013). Statistical notes for clinical researchers: Assessing normal distribution (2) using skewness and kurtosis. *Restorative Dentistry & Endodontics*, *38*(1), 52. https://doi.org/10.5395/rde.2013.38.1.52

Marsh, H. W., Martin, A. J., & Cheng, J. H. S. (2008). A multilevel perspective on gender in classroom motivation and climate: Potential benefits of male teachers for boys? *Journal of Educational Psychology*, *100*(1), 78–95. https://doi.org/10.1037/0022-0663.100.1.78

Martin, A. J., & Marsh, H. (2005). Motivating boys and motivating girls: Does teacher gender really make a difference? *Australian Journal of Education*, *49*(3), 320–334. https://doi.org/10.1177/000494410504900308

*Copyright © 2022 Nagy, Martin and Collie. This is an open-access article distributed under the terms of the Creative Commons Attribution License (CC BY). The use, distribution or reproduction in other forums is permitted, provided the original authors and copyright owners are credited and that the original publication in this journal is cited, in accordance with accepted academic practice. No use, distribution or reproduction is permitted which does not comply with these terms.*

**Table S1**

*Nine-item Student-rated Effort Scale*

| | |
|---|---|
| *Operative Effort Items* | I try hard on my schoolwork (e.g., in class or at home etc.) given to me<br>I do schoolwork (e.g., in class or at home etc.) to the best of my ability<br>I complete schoolwork (e.g., in class or at home etc.) by the due date |
| *Cognitive Effort Items* | I concentrate during class<br>I am focused in class<br>I pay attention in class |
| *Social-Emotional Effort Items* | I show self-control in lessons (e.g., I wait my turn, don't interrupt, don't talk over other students etc.)<br>I show respect to the teacher<br>I show respect to other students |

*Notes:* All items were rated using a 7-point Likert scale (1 = *strongly disagree t*o 7 = *strongly agree*), and were prefaced with the words "In mathematics … ". It is recommended that similar wording is used in future surveys to ensure the classroom specificity of the survey (e.g., "In this class, …").

**Table S2**

*Single-Level (L1) Descriptive Statistics and CFAs of 1) First-Order Effort 2) Higher-Order Effort and 3) Short-Scale Effort using Effort Scale – Short (ES-S) Items*

| Variable | Statistics | | | | | |
|---|---|---|---|---|---|---|
| | *M* | *SD* | Skew | Kurtosis | ω | CFA loadings (min., max., mean) |
| Level 1 (Student) | | | | | | |
| *CFA[1] of First-order effort factors* | | | | | | |
| Operative effort | 5.934 | 0.955 | -1.362 | 2.755 | .862 | .702, .878, .819 |
| Cognitive effort | 5.779 | 1.051 | -1.471 | 3.225 | .932 | .846, .939, .905 |
| Social-emotional effort | 6.243 | 0.713 | -1.102 | 1.915 | .783 | .694, .774, .739 |
| *CFA[2] of Second-order effort factor* | | | | | | |
| Higher-order effort | 5.985 | 0.797 | -1.093 | 1.733 | .891 | .697, .965, .850 |
| *CFA[3] of Effort Scale - Short factor* | | | | | | |
| Short-scale effort | 5.761 | 0.963 | -1.200 | 2.298 | .809 | .615, .851, .761 |

*Notes.* [1] First-order CFA model; [2] Higher-order CFA model; [3] CFA model using Effort Scale – Short (ES-S) items; ω = reliability (omega total; McNeish, 2018); CFA Loadings = Confirmatory factor analysis standardized factor loadings; *M*, *SD*, Skew and Kurtosis are calculated from unit-weighted scale scores of raw items.

**Table S3**

*Three-item Student-rated Effort Scale - Short (ES-S)*

| | |
|---|---|
| *Operative Effort Item* | I try to the best of my ability on schoolwork (in class or at home etc.) |
| *Cognitive Effort Item* | I focus well, stay on task, and pay attention |
| *Social-Emotional Effort Item* | I show self-control and respect to others (e.g., I wait my turn, don't interrupt etc.) |

*Notes:* All items were rated using a 7-point Likert scale (1 = *strongly disagree t*o 7 = *strongly agree*), and were prefaced with the words "In mathematics … ". It is recommended that similar wording is used in future surveys to ensure the classroom specificity of the survey (e.g., "In this class, …").

*Copyright © 2022 Nagy, Martin and Collie. This is an open-access article distributed under the terms of the Creative Commons Attribution License (CC BY). The use, distribution or reproduction in other forums is permitted, provided the original authors and copyright owners are credited and that the original publication in this journal is cited, in accordance with accepted academic practice. No use, distribution or reproduction is permitted which does not comply with these terms.*

**Table S4**

*Invariance Tests - Model Fit Measures for 3-Factor CFA Analyses –First Order Effort*

| | $\chi^2$ | *df* | RMSEA | CFI | ΔRMSEA | ΔCFI |
|---|---|---|---|---|---|---|
| Invariance tests – Gender | | | | | | |
| All parameters free (no invariance) – Configural Model | 136.83 | 54 | .057 | .969 | – | – |
| Loadings constrained as invariant – Metric Model | 141.68 | 60 | .054 | .970 | -.003 | .001 |
| Invariance tests – Age (3-Groups[a]) | | | | | | |
| All parameters free (no invariance) – Configural Model | 190.06 | 84 | .063 | .961 | – | – |
| Loadings constrained as invariant – Metric Model | 186.62 | 96 | .055 | .966 | -.008 | .005 |
| Invariance tests – NESB | | | | | | |
| All parameters free (no invariance) – Configural Model | 169.40 | 54 | .067 | .957 | – | – |
| Loadings constrained as invariant – Metric Model | 164.13 | 60 | .061 | .961 | -.006 | .004 |
| Invariance tests – SES (2-Groups) | | | | | | |
| All parameters free (no invariance) – Configural Model | 146.30 | 54 | .061 | .967 | – | – |
| Loadings constrained as invariant – Metric Model | 152.18 | 60 | .058 | .967 | -.003 | .000 |

*Note.* $\chi^2$ = Chi-squared value; *df* = Degrees of Freedom; ΔRMSEA = change in RMSEA (significant if > .015); ΔCFI = change in CFI (significant if < -.01). [a] Three groups were chosen to create approximately equal proportions (24%, 37%, 39%). The test was also invariant using two (unequal) groups.

**Table S5**

*Invariance Tests - Model Fit Measures for 4-Factor CFA Analyses –Higher Order Effort*

| | $\chi^2$ | *df* | RMSEA | CFI | ΔRMSEA | ΔCFI |
|---|---|---|---|---|---|---|
| Invariance tests – Gender | | | | | | |
| All parameters free (no invariance) – Configural Model | 129.63 | 48 | .060 | .970 | – | – |
| Loadings constrained as invariant – Metric Model | 135.93 | 56 | .055 | .970 | -.005 | .000 |
| Invariance tests – Age (3-Groups[a]) | | | | | | |
| All parameters free (no invariance) – Configural Model | 178.25 | 72 | .068 | .961 | – | – |
| Loadings constrained as invariant – Metric Model | 174.18 | 88 | .056 | .968 | -.012 | .007 |
| Invariance tests – NESB | | | | | | |
| All parameters free (no invariance) – Configural Model | 161.67 | 48 | .071 | .958 | – | – |
| Loadings constrained as invariant – Metric Model | 159.84 | 56 | .063 | .961 | -.008 | .003 |
| Invariance tests – SES (2-Groups) | | | | | | |
| All parameters free (no invariance) – Configural Model | 139.02 | 48 | .065 | .967 | – | – |
| Loadings constrained as invariant – Metric Model | 155.05 | 56 | .062 | .964 | -.003 | -.003 |

*Note.* $\chi^2$ = Chi-squared value; *df* = Degrees of Freedom; ΔRMSEA = change in RMSEA (significant if > .015); ΔCFI = change in CFI (significant if < -.01). [a] Three groups were chosen to create approximately equal proportions (24%, 37%, 39%). The test was also invariant using two (unequal) groups.

*Copyright © 2022 Nagy, Martin and Collie. This is an open-access article distributed under the terms of the Creative Commons Attribution License (CC BY). The use, distribution or reproduction in other forums is permitted, provided the original authors and copyright owners are credited and that the original publication in this journal is cited, in accordance with accepted academic practice. No use, distribution or reproduction is permitted which does not comply with these terms.*

**Table S6**

*Invariance Tests - Model Fit Measures for 1-Factor CFA Analyses –Effort Scale – Short (ES-S)*

| | $\chi^2$ | *df* | RMSEA | CFI | ΔRMSEA | ΔCFI |
|---|---|---|---|---|---|---|
| Invariance tests – Gender | | | | | | |
| All parameters free (no invariance) – Configural Model | 44.21 | 2 | .212 | .895 | – | – |
| Loadings constrained as invariant – Metric Model | 31.43 | 4 | .121 | .932 | -.091 | .037 |
| Invariance tests – Age (3-Groups[a]) | | | | | | |
| All parameters free (no invariance) – Configural Model | 16.56 | 4 | .100 | .968 | – | – |
| Loadings constrained as invariant – Metric Model | 11.92 | 8 | .039 | .990 | -.061 | .022 |
| Invariance tests – NESB | | | | | | |
| All parameters free (no invariance) – Configural Model | 12.72 | 2 | .106 | .975 | – | – |
| Loadings constrained as invariant – Metric Model | 10.83 | 4 | .060 | .984 | -.046 | .009 |
| Invariance tests – SES (2-Groups) | | | | | | |
| All parameters free (no invariance) – Configural Model | 6.74 | 2 | .072 | .990 | – | – |
| Loadings constrained as invariant – Metric Model | 9.50 | 4 | .055 | .988 | -.017 | -.002 |

*Note.* $\chi^2$ = Chi-squared value; *df* = Degrees of Freedom; ΔRMSEA = change in RMSEA (significant if > .015); ΔCFI = change in CFI (significant if < -.01). [a] Three groups were chosen to create approximately equal proportions (24%, 37%, 39%). The test was also invariant using two (unequal) groups.

*Copyright © 2022 Nagy, Martin and Collie. This is an open-access article distributed under the terms of the Creative Commons Attribution License (CC BY). The use, distribution or reproduction in other forums is permitted, provided the original authors and copyright owners are credited and that the original publication in this journal is cited, in accordance with accepted academic practice. No use, distribution or reproduction is permitted which does not comply with these terms.*

**Table S7**

*Multilevel Structural Equation Process Model MSEM$_2$: Standardized Beta Coefficients – Higher-Order Effort Factor*

| Variables | Outcomes | | | | | | |
|---|---|---|---|---|---|---|---|
| | Self-efficacy | Valuing | Mastery orientation | Anxiety | Failure avoidance | Uncertain control | Higher-order effort |
| Level 1 (Student) | | | | | | | |
| *L1 Covariates* | | | | | | | |
| SES | .053 | .033 | .008 | .064 | .013 | .013 | -.010 |
| Age | .022 | .034 | .028 | -.032 | -.025 | -.014 | -.016 |
| Gender (male) | .160*** | .102** | .041 | -.161*** | -.033 | -.083** | -.087* |
| NESB | -.045 | .053 | -.002 | -.026 | .060 | .069 | -.036 |
| Mathematics ability | .213*** | .160*** | .044 | -.070 | -.123** | -.142** | .061* |
| *L1 Motivation factors* | | | | | | | |
| Self-efficacy (positive) | | | | | | | .272*** |
| Valuing (positive) | | | | | | | .198** |
| Mastery orientation (positive) | | | | | | | .295*** |
| Anxiety (negative) | | | | | | | .138** |
| Failure avoidance (negative) | | | | | | | .005 |
| Uncertain control (negative) | | | | | | | -.113* |
| Level 2 (Classroom) | | | | | | | |
| *L2 Covariates* | | | | | | | |
| Class-average ability | .368** | .437** | -.127 | -.097 | -.550*** | -.629*** | .238 |
| Class size | .298* | -.008 | .379* | .292 | .407* | -.142 | -.171 |
| Class-average age | -.153 | -.361*** | -.287** | .204 | -.036 | -.101 | -.135 |
| *L2 Motivation factors* | | | | | | | |
| Self-efficacy (positive) | | | | | | | .292 |
| Valuing (positive) | | | | | | | .149 |
| Mastery orientation (positive) | | | | | | | .423** |
| Anxiety (negative) | | | | | | | .101 |
| Failure avoidance (negative) | | | | | | | .027 |
| Uncertain control (negative) | | | | | | | -.148 |

*Notes.* Motivation items modelled as error-adjusted scores. SES = Social-economic status indicator (positive is higher SES); NESB = Non-English speaking background.
$^{*}$ $p < .05$. $^{**}$ $p < .01$. $^{***}$ $p < .001$.

*Copyright © 2022 Nagy, Martin and Collie. This is an open-access article distributed under the terms of the Creative Commons Attribution License (CC BY). The use, distribution or reproduction in other forums is permitted, provided the original authors and copyright owners are credited and that the original publication in this journal is cited, in accordance with accepted academic practice. No use, distribution or reproduction is permitted which does not comply with these terms.*

**Table S8**

*Multilevel Descriptive Statistics and CFA of Effort Scale - Short (ES-S) and Motivation*

| Variable | Statistics | | | | | | |
|---|---|---|---|---|---|---|---|
| | *M* | *SD* | Skew | Kurtosis | ω[a] | CFA loadings (min., max., mean) | ICC |
| Level 1 (Student) | | | | | | | |
| Short-scale effort | 5.761 | 0.963 | -1.200 | 2.298 | .776 | .565, .810, .728 | - |
| Self-efficacy (positive motivation) | 5.822 | 1.333 | -1.683 | 2.950 | .771 | .852 | - |
| Valuing (positive motivation) | 5.542 | 1.384 | -1.120 | 1.018 | .770 | .847 | - |
| Mastery orientation (positive motivation) | 5.590 | 1.283 | -1.145 | 1.329 | .806 | .888 | - |
| Anxiety (negative motivation) | 5.251 | 1.754 | -0.923 | -0.129 | .771 | .759 | - |
| Failure avoidance (negative motivation) | 4.715 | 1.819 | -0.474 | -0.862 | .766 | .765 | - |
| Uncertain control (negative motivation) | 3.012 | 1.665 | 0.679 | -0.439 | .788 | .821 | - |
| Level 2 (Classroom) | | | | | | | |
| Short-scale effort | 5.717 | 0.498 | -0.930 | 0.805 | .946 | .812, .995, .921 | .240 |
| Self-efficacy (positive motivation) | 5.738 | 0.635 | -0.986 | 0.582 | .777 | .978 | .183 |
| Valuing (positive motivation) | 5.507 | 0.619 | -0.578 | 0.871 | .789 | .971 | .165 |
| Mastery orientation (positive motivation) | 5.570 | 0.445 | -0.709 | 0.595 | .840 | .969 | .106 |
| Anxiety (negative motivation) | 5.210 | 0.493 | -0.260 | -0.203 | .779 | .936 | .072 |
| Failure avoidance (negative motivation) | 4.746 | 0.605 | -0.143 | 0.428 | .842 | .963 | .098 |
| Uncertain control (negative motivation) | 3.120 | 0.680 | 0.095 | -0.201 | .876 | .980 | .141 |

*Notes.* ω = reliability (omega total; McNeish, 2018); ICC = Intra Class Correlation; CFA Loadings = Confirmatory factor analysis standardized factor loadings; *M*, *SD*, Skew and Kurtosis are calculated from unit-weighted scale scores of raw items. [a] Motivation items are modelled as error-adjusted scores using established reliability and variance measures from a prior research program (ω and $\sigma^2$ values were derived from data used in: Marsh et al., 2008; Martin & Marsh, 2005).

*Copyright © 2022 Nagy, Martin and Collie. This is an open-access article distributed under the terms of the Creative Commons Attribution License (CC BY). The use, distribution or reproduction in other forums is permitted, provided the original authors and copyright owners are credited and that the original publication in this journal is cited, in accordance with accepted academic practice. No use, distribution or reproduction is permitted which does not comply with these terms.*

**Table S9**

*Multilevel Correlation Matrix using Effort Scale - Short (ES-S) within and between Motivation and Effort factors*

| Variables | Effort | Self-efficacy | Valuing | Mastery orientation | Anxiety | Failure avoidance |
|---|---|---|---|---|---|---|
| Level 1 (Student) | | | | | | |
| *Motivation factors* | | | | | | |
| Self-efficacy (positive) | .586*** | | | | | |
| Valuing (positive) | .603*** | .675*** | | | | |
| Mastery orientation (positive) | .584*** | .407*** | .532*** | | | |
| Anxiety (negative) | .033 | -.158** | -.058 | -.002 | | |
| Failure avoidance (negative) | -.063 | -.176*** | -.191*** | -.030 | .537*** | |
| Uncertain control (negative) | -.266*** | -.497*** | -.329*** | -.181*** | .370*** | .387 |
| Level 2 (Classroom) | | | | | | |
| *Motivation factors* | | | | | | |
| Self-efficacy (positive) | .782*** | | | | | |
| Valuing (positive) | .791*** | .770*** | | | | |
| Mastery orientation (positive) | .782*** | .631*** | .683*** | | | |
| Anxiety (negative) | .152 | .078 | .044 | .016 | | |
| Failure avoidance (negative) | -.015 | -.014 | -.043 | .180 | .332** | |
| Uncertain control (negative) | -.561*** | -.697*** | -.527*** | -.243 | .062 | .301* |

*Notes.* Motivation items are modelled as error-adjusted scores; * $p < .05$. ** $p < .01$. *** $p < .001$.

*Copyright © 2022 Nagy, Martin and Collie. This is an open-access article distributed under the terms of the Creative Commons Attribution License (CC BY). The use, distribution or reproduction in other forums is permitted, provided the original authors and copyright owners are credited and that the original publication in this journal is cited, in accordance with accepted academic practice. No use, distribution or reproduction is permitted which does not comply with these terms.*

**Table S10**

*Multilevel Structural Equation Process Model using Effort Scale – Short (ES-S): Standardized Beta Coefficients*

| Variables | Outcomes | | | | | | |
|---|---|---|---|---|---|---|---|
| | Self-efficacy | Valuing | Mastery orientation | Anxiety | Failure avoidance | Uncertain control | Effort |
| Level 1 (Student) | | | | | | | |
| *L1 Covariates* | | | | | | | |
| SES | .054 | .034 | .008 | .064 | .012 | .013 | .031 |
| Age | .022 | .034 | .028 | -.032 | -.025 | -.014 | -.023 |
| Gender (male) | .160*** | .102** | .041 | -.161*** | -.033 | -.083** | -.101* |
| NESB | -.045 | .052 | -.002 | -.026 | .060 | .069 | -.027 |
| Mathematics ability | .214*** | .160*** | .044 | -.070 | -.123** | -.142** | .073* |
| *L1 Motivation factors* | | | | | | | |
| Self-efficacy (positive) | | | | | | | .310*** |
| Valuing (positive) | | | | | | | .216* |
| Mastery orientation (positive) | | | | | | | .344*** |
| Anxiety (negative) | | | | | | | .078 |
| Failure avoidance (negative) | | | | | | | .011 |
| Uncertain control (negative) | | | | | | | -.007 |
| Level 2 (Classroom) | | | | | | | |
| *L2 Covariates* | | | | | | | |
| Class-average ability | .368** | .437** | -.127 | -.097 | -.550*** | -.629*** | .178 |
| Class size | .298* | -.008 | .379* | .292 | .407* | -.142 | -.140 |
| Class-average age | -.153 | -.361*** | -.287** | .204 | -.036 | -.101 | -.079 |
| *L2 Motivation factors* | | | | | | | |
| Self-efficacy (positive) | | | | | | | .114 |
| Valuing (positive) | | | | | | | .158 |
| Mastery orientation (positive) | | | | | | | .537*** |
| Anxiety (negative) | | | | | | | .130 |
| Failure avoidance (negative) | | | | | | | -.019 |
| Uncertain control (negative) | | | | | | | -.233 |

*Notes.* Motivation items modelled as error-adjusted scores. SES = Social-economic status indicator (positive is higher SES); NESB = Non-English speaking background; $^{*}$ $p < .05$. $^{**}$ $p < .01$. $^{***}$ $p < .00$

*Copyright © 2022 Nagy, Martin and Collie. This is an open-access article distributed under the terms of the Creative Commons Attribution License (CC BY). The use, distribution or reproduction in other forums is permitted, provided the original authors and copyright owners are credited and that the original publication in this journal is cited, in accordance with accepted academic practice. No use, distribution or reproduction is permitted which does not comply with these terms.*
